# Pathways of Parental Education on Children's and Adolescent's Body Mass Index: The Mediating Roles of Behavioral and Psychological Factors

**DOI:** 10.3389/fpubh.2022.763789

**Published:** 2022-03-07

**Authors:** Teresa Seum, Ann-Katrin Meyrose, Matthias Rabel, Anja Schienkiewitz, Ulrike Ravens-Sieberer

**Affiliations:** ^1^Department of Child and Adolescent Psychiatry, Psychotherapy, and Psychosomatics, University Medical Center Hamburg-Eppendorf, Hamburg, Germany; ^2^Clinical Psychology, Helmut-Schmidt-University/University of the Federal Armed Forces, Hamburg, Germany; ^3^Department of Sport and Health Sciences, Technical University of Munich, Munich, Germany; ^4^Department of Epidemiology and Health Monitoring, Robert Koch-Institute, Berlin, Germany

**Keywords:** BMI, BELLA study, mediation analysis, longitudinal study, youth, breakfast consumption, screen time

## Abstract

**Aim:**

The increasing body mass index (BMI) often followed by overweight and obesity is a global health problem of the 21st century. Children and adolescents with lower socioeconomic status are more affected than their counterparts. The mechanisms behind these differences must be well understood to develop effective prevention strategies. This analysis aims at examining the association of parental education as an indicator of the socioeconomic status on children's and adolescent's body mass index and the role of behavioral and psychological risk factors for a higher BMI longitudinally.

**Methods:**

The analysis was based on a nationwide sample of *N* = 460 children and adolescents, aged 11 to 17 at baseline (2009–2012), who took part in the representative BELLA study, the mental health module of the German National Health Interview and Examination Survey among Children and Adolescents (KiGGS). A follow-up was conducted 5 years later. Using mediation analyses, the mediating effects of breakfast consumption, consumption of sugar-sweetened beverages, screen time, physical activity, mental health problems (Strengths and Difficulties Questionnaire), and health-related quality of life (KIDSCREEN-10) on the association of parent's years of education on their children's BMI were investigated.

**Results:**

A lower level of parental education was significantly associated with a higher BMI in children and adolescents 5 years later. The association was partially mediated by breakfast consumption and total screen time, with breakfast consumption mediating 16.7% and total screen time 27.8% of the association. After controlling for age, gender, and migration status, only breakfast consumption remained a partial mediator (8.5%). Other included variables had no mediating effects.

**Conclusions:**

Preventive measures should be mainly targeted at children and adolescents of parents with lower educational levels. Tailored strategies to prevent the development of overweight and obesity in this population among children and adolescents should promote daily breakfast consumption at home and reducing screen time.

## Introduction

The increasing body mass index (BMI) often followed by overweight and obesity is one of the most significant challenges facing public health in the 21st century (1). Worldwide more than 340 million children and adolescents aged 5 to 19 were affected by overweight or obesity in year 2016; thus, 11.4% of children and adolescents had overweight, and 6.6% had obesity (2). The current prevalence in Germany is also considerable, with 15.4% of children and adolescents having overweight and 5.9% having obesity (3). An increased BMI, overweight and obesity have serious consequences for children and adolescents (1). It can have a significant adverse effect on physical health as well as on social and emotional wellbeing and self-esteem (4, 5). Furthermore, childhood obesity is associated with a lower quality of life, poor school performance of children, and elevated risk of teasing, bullying, and social isolation (1, 4). Children and adolescents with an increased BMI, are at higher risk of developing overweight and obesity and subsequently, diseases such as cardiovascular disease, hypertension, type 2 diabetes, osteoarthritis, respiratory diseases, and certain cancers (including colorectal cancer, kidney cancer, and esophageal cancer) (6–8). The negative consequences are usually also observed in adulthood as many children with a high BMI, overweight or obesity maintain their high weight as an adult (9). Long-term effects are premature mortality, a significantly increased risk of later cardiometabolic morbidity, and a significantly increased risk of disability pension (10, 11). In addition, childhood obesity also has many economic consequences (12, 13). The high prevalence, the complex influencing factors, and the severe consequences make overweight and obesity a significant challenge for the health care system in various areas worldwide.

Overweight and obesity are considered to be the result of a complex interplay of genetic, hormonal and nutritional influences, physical activity as well as social and environmental factors (14). However, overweight and obesity and their related diseases are largely preventable through early and effective prevention (15). Particularly modifiable factors, such as environmental and behavioral factors, are essential in the prevention of overweight and obesity and should, therefore, be given special attention (16). Studies have shown that various factors in prevention, such as limited consumption of sugar-sweetened beverages, daily breakfast, reduced screen time, and increased physical activity, are essential in preventing childhood obesity (17–19). Moreover, support for mental health problems and the improvement of quality of life also contribute to preventing overweight (20, 21) as well as the consideration of family factors such as socioeconomic status (SES) (22).

Numerous studies suggest that SES affects the risk of developing obesity in both adults and children (23–25). For Germany, the inverse relationship was confirmed by the representative study for children and youth health, KiGGS. The prevalence of overweight was 22.5% for low SES, 17.4% for medium SES, and only 12.8% for high SES (26).

Parental education, as an indicator of SES, has the most consistent inverse association with childhood obesity (24, 27, 28). This may partly be due to the fact that parental education has the advantage of being relatively stable; it does not fluctuate according to transient life events as income or occupation can (29). However, the parental educational level does not directly affect the development of overweight or obesity of children, but moreover the behavior and lifestyle factors leading to overweight and obesity (30–32). A higher parental educational level can promote the parents' ability to process health information, which may lead to improved health-related decisions in parenting practice and can also influence the parents' motivation to practice a healthy lifestyle as a role model for their children (33, 34). The pathways from SES, measured by the indicators parental education, occupational status, and household income, to health and health-related behaviors were recently analyzed in the KiGGS study (35, 36). Results of the study show significant inequalities to the detriment of adolescents from families with low SES in the areas of mental health, physical activity, fresh fruit intake, consumption of sugar-sweetened beverages, and smoking (35, 36). Most of these factors can also be associated with the body mass index of children and adolescents (20, 37–40).

To better understand the pathways between parental education, health-related and psychological factors, and body mass index of children and adolescents, mediation analyses are needed. Some cross-sectional studies have already investigated the relationship between parental education, health-related behaviors, and body composition (41–43). The “EuropeaN Energy balance Research to prevent excessive weight Gain among Youth” (ENERGY) project detected that the path between parental education and children's body composition was partially mediated by breakfast consumption, sugar-sweetened beverage consumption, TV viewing, computer use, and sports participation (41). A study by Manios et al. (42) showed that the lower likelihood of having overweight or obesity and higher parental educational status was partially mediated by children's daily breakfast consumption. A study in the Netherlands identified watching TV and consuming breakfast as contributing factors in this association (43). Studies are still lacking that investigate direct and indirect relationships at a longitudinally level as well as studies including psychological factors as mediators (44).

To fill this research gap and therefore better understand the pathways to obesity, develop prevention and intervention programs, and support effective policy-making, this study aimed to investigate the direct and indirect effects of parental education via behavioral and psychological factors on the body mass index of children and adolescents aged 11 to 17 years longitudinally. Single mediation analyses were used to individually assess the effect of parental education via behavioral and psychological factors (breakfast consumption, consumption of sugar-sweetened beverages, total screen time, physical activity, mental health problems and quality of life) on the body mass index. Multiple mediation analyses were carried out to compare the importance of the mediators. Age, gender, and migration status were included in the analyses as covariates. They have shown to be significantly linked to the outcome and the mediators (45). This study used data from the nationally representative survey on mental health and behavior of children and adolescents in Germany, the BELLA study (46).

In this study we focused on the following four hypotheses: (1) a lower level of parental education is associated with a higher body mass index in children and adolescents 5 years later; (2) a lower level of parental education is linked to behavioral and psychological risk factors (skipping breakfast, higher consumption of sugar-sweetened beverages, higher amount of total screen time, lower physical activity, higher amount of mental health problems and lower quality of life); (3) more pronounced risk factors cohere with a higher body mass index 5 years later; (4) parental education has an indirect effect (via breakfast consumption, consumption of sugar-sweetened beverages, total screen time, physical activity, mental health problems, quality of life) on the child's and adolescent's body mass index.

## Materials and Methods

### Study Design

The data for the following analyses comes from the prospective, longitudinal BELLA cohort study, the mental health module of the German National Health Interview and Examination Survey for children and adolescents (KiGGS) (46, 47). The BELLA cohort study is a representative subsample of KiGGS and examines the mental health and wellbeing of children and adolescents aged 7 to 17 years. Study participants were enrolled in a two-stage random sampling procedure, consisting of a selection of 167 sample points throughout Germany and further sampling of participants from official registers of the residents' registration offices. Data from the baseline assessment (2009–2012) and the follow-up (2014–2017) were used in the analyses for this study. Through computer-assisted telephone interviews and subsequent questionnaires, data was collected for the baseline assessment. For the follow-up, participants were additionally examined by a medical professional. Before being questioned or examined, written informed consent was given by the participants' parents (if aged 7–17) and additionally by the participants themselves (aged 14 or older) for each measurement point.

The BELLA study was approved for both measurement points from the ethics committee of the University Hospital Charitè in Berlin, and the Federal Commissioner for Data Protection in Germany. Design and methods are published elsewhere (46, 48).

### Participants

**Figure 1:**
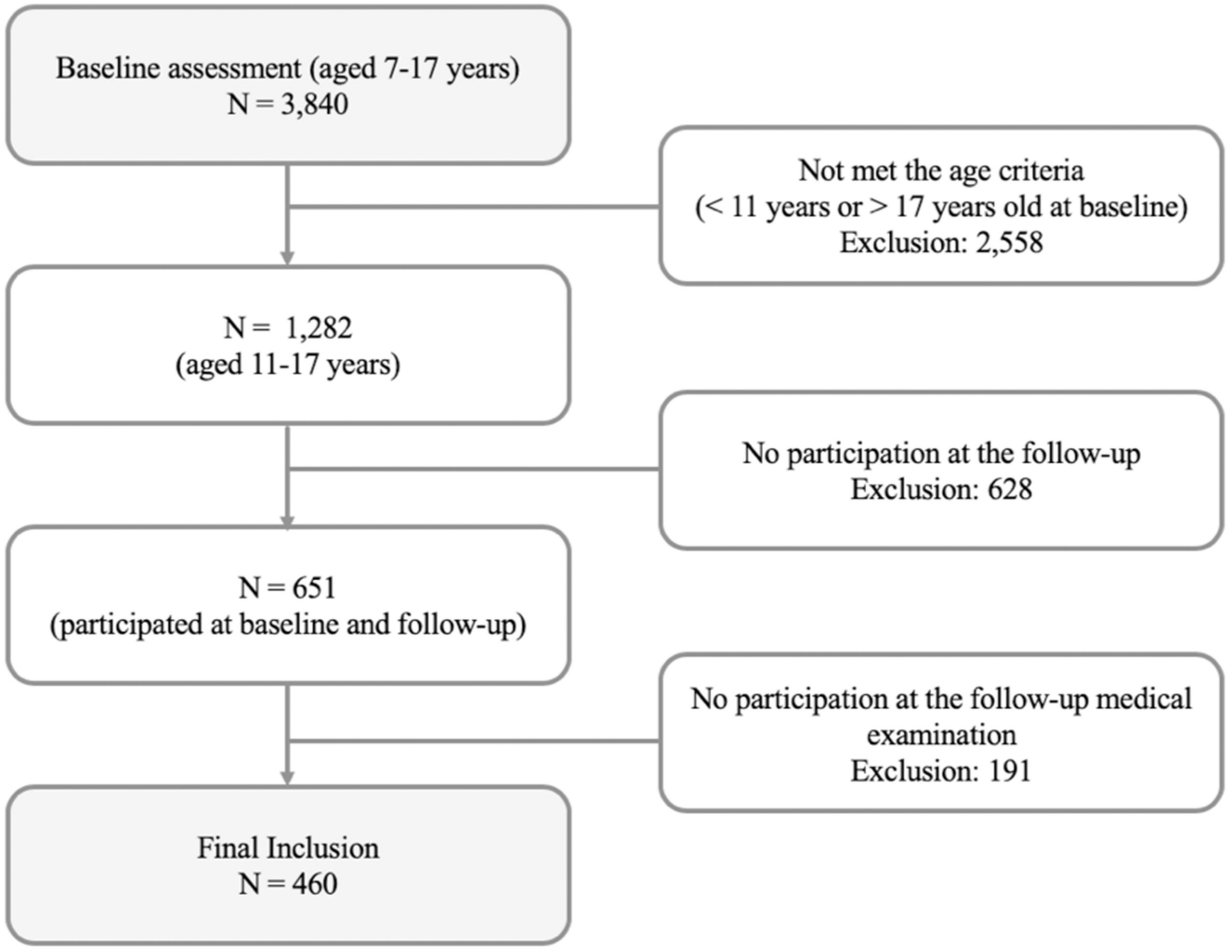
Flow chart for selection of study participants based on the prerequisites for inclusion.

Of 3,840 children and adolescents partaking in the BELLA study baseline assessment, 460 participants were included in the presented analyses. Information about general drop-out and response in the BELLA study can be found in Otto et al. (46). The prerequisites for inclusion in the study were the following: (i) participation at the baseline assessment, (ii) age between 11 and 17 at baseline, (iii) participation at the follow-up and (iv) participation at the physical examination at the follow-up. Age restriction is caused due to the need for both parent-reported and self-reported data at the baseline assessment. Baseline characteristics of the study sample and children and adolescents of the same age range without follow-up data were compared. The selection of study participants is also presented in Figure 1.

### Measurements

#### Outcome Measure: Children's and Adolescent's Body Mass Index

Standardized measurements of body weight and height were obtained at the physical examination at the follow-up. Participants were measured in underwear by trained assistants. For each of the participants, the body mass index (BMI) was calculated as weight in kilograms divided by height in meters squared. For sensitivity analysis, we utilized BMI percentile ranks to allow comparisons across age and gender, since they are less likely to be outlier prone than raw BMI. The percentile curves based on the German population by Kromeyer-Hauschild et al. (49) were used as the reference population. In addition to data for adolescents, percentiles for adults were included in the analyses to avoid methodological changes in the transition from adolescence to early adulthood (49).

BMI was classified according to international age- and gender-specific International Obesity Task Force (IOTF) standardized BMI cut-off points for participants under the age of 18 (50). The BMI of participants aged 18 and over was categorized using the World Health Organization guidelines (51). According to the guidelines, underweight was defined as a BMI <18.5, normal weight as 18.5 ≤ BMI <25, overweight as 25 ≤ BMI <30, and obesity as a BMI ≥ 30.

#### Predictor: Parental Education

*Parental education* was measured at baseline assessment and reported by the parents. It consists of the sum of years spent on school education and vocational qualification. By using categories of German school-leaving certificates the parent's years spent on school education were estimated (e.g., 13 years for German *Abitur*; 10 years for German *Mittlere Reife*; 8 years for people still enrolled in school). In addition, their vocational qualifications were considered (e.g., 5 years for a university degree; 3 years for a completed vocational training; 1.5 years for a completed basic training). To calculate the total score of parental education, the mean of the mother's and father's years spent in school education and vocational qualification was added. The rational for using the average approach of both parents' education follows Thaning and Hällsten (52): Based on their analyses, authors recommend to use the averaged parental education for a simple interpretation prior to the conventional dominance approach or only information for one parent, because both parents are important and increase variation of socio-economic background.

#### Potential Mediators

Six potential mediators were used in the analyses; four behavioral factors, and two psychological factors. Behavioral factors were breakfast consumption, consumption of sugar-sweetened beverages, physical activity, and total screen time. Psychological factors were mental health problems and quality of life. All mediators were assessed at the baseline assessment, and all were self-reported.

##### Breakfast Consumption

To assess the participant's *breakfast consumption*, they were asked about on how many weekdays they eat breakfast at home. Weekdays were defined as Monday to Friday. Possible responses were scored the following: 5 = *Yes, always*, 3.5 = *Yes, 3 to 4 times a week*, 1.5 = *Yes, 1 to 2 times a week*, 0 = *Never*.

##### Consumption of Sugar-Sweetened Beverages

To determine the *consumption of sugar-sweetened beverages*, participants were asked the following questions: “How often do you drink sugar sweetened-beverages?.” Four answer possibilities were given: *Every day, At least once a week, Less than once a week* and *Never*. Depending on the answer, participants were asked: “How many glasses of sugar sweetened-beverages do you drink per day?” or “per week?.” A glass was defined as 200 ml. If the participant indicated a daily consumption in the first question, the portion was converted into the corresponding amount per week. A total score was calculated according to the frequency of consumption per week.

##### Physical Activity

Participants were asked as follows to assess *physical activity*: “On how many days of a normal week are you physically active for at least 60 min a day?.” The eight answer categories ranged from 0 = *On no day* to 7 = *7 days*.

##### Total Screen Time

To measure the *total amount of screen time* participants were asked: “How much time do you spend on average per day doing the following? Watching television/video, using a computer/internet, playing video games, using a cell phone.” Answer options were scored 0 = *not at all, 1* = *up to 1 h, 2* = *up to 2, 3* = *up to 3 h, 4* = *up to 4 h* and 5 = *more than 4 h*. A total screen time score was calculated by adding the hours spent on a daily basis with the respective activities and converting them into the amount per week.

##### Mental Health Problems

The Strength and Difficulty Questionnaire (SDQ) was used to assess *mental health problems* (53). The Total Difficulties Score was calculated by summing 20 items from the four subscales “emotional problems,” “conduct problems,” “hyperactivity/inattention,” and “peer relationship problems.” It ranges from 0 to 40, with higher score indicating more serious mental health problems. The items of the SDQ were scored on a three-point scale (1 = *not true*, 2 = *somewhat true, 3* = *certainly true*). The SDQ is a valid and reliable questionnaire which is well-established for the screening of mental health problems in children and adolescents (54).

##### Health-Related Quality of Life

Using the KIDSCREEN-10, the *health-related quality of life* (HRQoL) of the participants was assessed (55). The KIDSCREEN-10 is a brief form of the KIDSCREEN-52 and measures HRQoL from the child's perspective with 10 items. Responses are recorded on a 5-point response scale ranging from *never* to *always*, or from *not at all* to *extremely*. Items were reversed where necessary to ensure that higher scores indicate a better HRQoL. For each participant, items were summed and were transformed into *T*-values with a mean of 50 and a standard deviation of approximately 10. Previous studies have established that this index has acceptable reliability, with a Cronbach's alpha > 0.80 (56, 57).

#### Covariates (Age, Gender, Migration Background)

Covariates were assessed at baseline and included the participant's gender (0 = *female*, 1 = *male*), their age (in years) and their migration status (0 = *no*, 1 = *yes*). A child or adolescent was defined as having a migration status if they immigrated from another country and at least one parent was not born in Germany, or both parents immigrated and/or had non-German citizenship.

### Statistical Analysis

**Figure 2:**
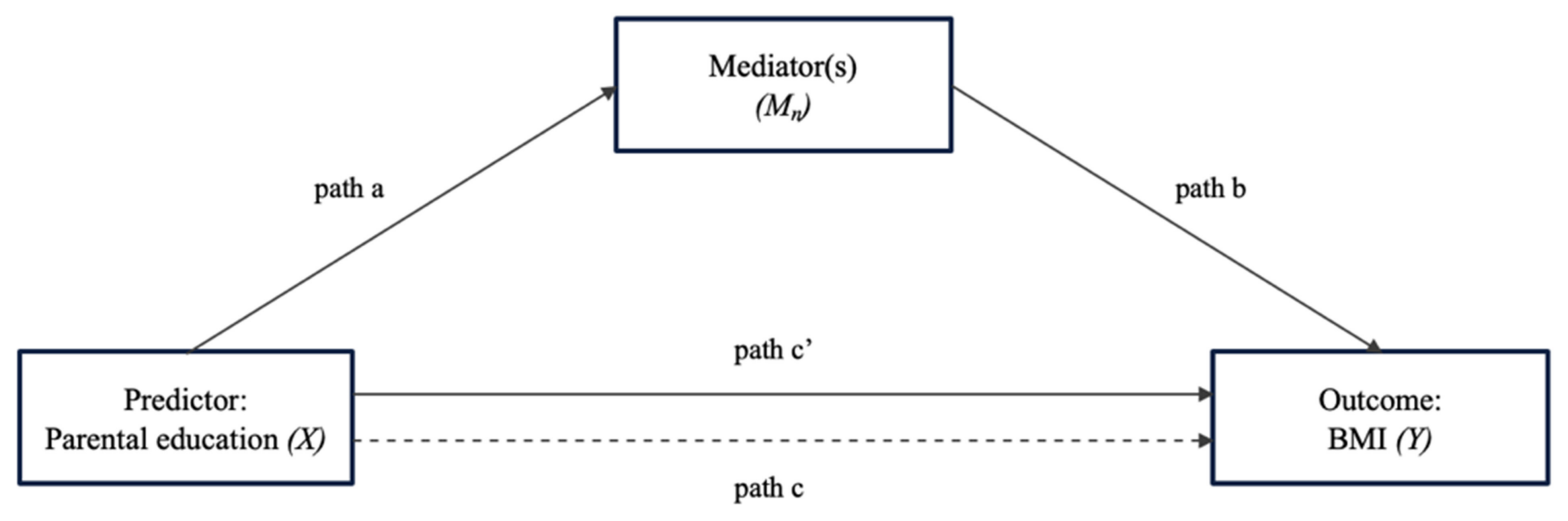
Path diagram for the effect of the predictor variable on the outcome variable through the mediator variable(s). a, effect of *X* on *M*; b, effect of *M* on *Y* controlling for the effect of *X*; c', direct effect of *X* on *Y*; c, total effect of *X* on *Y* (sum of the indirect effect and direct effect; i.e., *c* = a*b+*c*′).

Descriptive statistical analyses (i.e., frequencies, means, and standard deviations) were done for all analyzed variables. To explore whether breakfast consumption, consumption of sugar-sweetened beverages, physical activity, total screen time, mental health problems and HRQoL have an effect on the association between parental education and children's and adolescent's body mass index, mediation analyses were conducted. Mediation occurs when an independent variable (X) influences a dependent variable (Y) through one or more mediator(s) (M) (58, 59). The statistical analysis corresponds to the recommendations of Hayes (59). Figure 2 depicts the mediation model followed (PROCESS Macro Model 4).

First, each potential mediator (breakfast consumption, consumption of sugar-sweetened beverages, physical activity, total screen time, mental health problems and HRQoL) was examined separately in mediation analyses through PROCESS macro software on SPSS (60). In a single mediation analysis, path a represents the effect of X on M, and path b represents the effect of M on Y controlling for the effect of X. The direct effect of X on Y is expressed as path c′. The indirect effect of X on Y through M is the product of a and b (i.e., a^*^b). The total effect of X on Y is the sum of the indirect effect and direct effect (i.e., c = a^*^b +c′). Percentage mediated by the identified mediators is calculated by dividing the indirect effect by the total effect.

Afterward, the mediators with a significant indirect effect were included in the multiple mediator analyses. A multi mediator analysis includes two or more mediators simultaneously to compare the effects. Two multiple mediator analyses were conducted, one including the potential mediators (Multiple mediator model 1) and one adjusted for the covariates age, gender, interaction of age × gender and migration status (Multiple mediator model 2).

According to Baron and Kenny (61), to classify a variable as a mediator, the following conditions had to be met: (1) the predictor shows a significant effect on the mediator, (2) the predictor shows a significant effect on the outcome when the effect of the mediator is not controlled, (3) the mediator has a statistically significant effect on the outcome, and (4) the predictor's effect on the outcome decreases (partial mediation) or is no longer significant (complete mediation).

For the mediation analyses, the predictor parental education and all mediators as well as the covariate age were centered using the grand mean of the sample. For all analyses the effect sizes, *p*-values, and the corresponding 95% confidence intervals (CI) are reported. The mediated (indirect) effect was formally examined using a non-parametric bootstrapping procedure (*n* = 5,000 samples) that estimated the sampling distribution of the indirect effect and the corresponding bias-corrected and accelerated 95% CI (58, 59). Indirect effects were considered significant when the 95% CI did not include zero.

Prior to mediation model calculations, missing data of the predictor and mediators were replaced using the Expectation-Maximization (EM) algorithm in order to include all cases (*n* = 460). Missing values were below 5% for all mediators and the predictor, besides mental health problems (12%) and HRQoL (8%). However, missing cases did not show deviations from other cases. To compare results obtained using imputed data and complete data, sensitivity analyses were computed.

The mediation analyses were assessed using Version 3 of PROCESS macro software, which uses a regression-based approach to mediation (60). PROCESS allows in contrast to others direct quantification of the indirect effects. It uses bootstrapping, an alternative method for testing mediation, that does not assume normality of the sampling distribution and yields greater statistical power (62). This is particularly relevant for highly skewed outcome variables, such as BMI.

To conduct all analyses, version 23 of IBM SPSS was used. The significance level was set as α <0.05 for all analyses.

## Results

### Sample Characteristics

Table 1 presents descriptive statistics for all variables in the analyses. Longitudinal data of *n* = 460 of the children and adolescents (52.6% female) were analyzed. At the baseline assessment, participants were between 11 and 17 years old (*M* = 14.00, *SD* = 1.84) and between 15 and 23 years at the follow-up (*M* = 18.66, *SD* = 1.92). Overall, 6.5% of the participants had a migration status.

**Table 1:** Description of the study sample.

	**Children and adolescents** **(*****n*** **=** **460)**		
	***n***	**Valid %**	***M* (*SD*)**
Gender			
Male	218	47.4	
Female	242	52.6	
Age (in years)			
T0 (11–17 years)	460		14.00 (1.84)
T1 (15–23 years)	460		18.66 (1.92)
Migration status (T0)			
Yes	30	6.5	
No	430	93.5	
Parental education (in years, T0)	459		13.77 (2.34)
BMI (kg/m^2^) (T1)	460		22.61 (3.98)
BMI-categories			
Underweight	35	7.6	
Normal weight	328	71.3	
Overweight	74	16.1	
Obese	23	5.0	
Breakfast consumption (in days per weekdays, T0)	451		3.94 (1.76)
Sugar-sweetened beverages (in glasses per week, T0)	449		7.24 (14.74)
Total screen time (in hours per week, T0)	451		38.71 (20.81)
Physical activity (in days per week, T0)	449		3.93 (1.78)
Mental health problems (SDQ total score, T0)	405		9.47 (4.39)
HRQoL (KIDSCREEN-10, T0)	423		52.03 (8.72)

*T0, Baseline assessment; T1, Follow-up; SDQ, Strengths and Difficulties Questionnaire (51); KIDSCREEN-10, HRQOL Index (53). M, mean; SD, standard deviation*.

The mean BMI measured at the follow-up assessment was 22.61 (*SD* = 3.98). Of all participants, 7.6% were categorized as underweight, 71.3% as normal weight, 16.1% as overweight, and 5.0% as obese. The mean years of parental education were 13.77 (*SD* = 2.34), with the lowest being 9.75 years and the highest 18 years.

The comparison of baseline characteristics of the sample used in the analyses and children and adolescents of the same age range without follow-up data revealed a small to medium difference in the averaged age (study sample: *M* = 14.00 vs. sample without follow-up data: *M* = 14.76) and small differences in breakfast consumption and total screen time with healthier behavior in the study sample. No further differences between the samples were detected. Underlying statistics are provided in Supplementary Table 1.

### Mediation Analyses

#### Single Mediator Models

Table 2 presents the results of the single mediation analyses for each potential mediator of the association between parental education and children's and adolescent's BMI. A significant total effect (path c) of parental education on the child's and adolescent's BMI was found. Children of parents with a lower educational level were significantly more likely to have a higher BMI (ß:−0.25; CI:−0.40,−0.09).

**Table 2:** Single mediator models.

**(*n* = 460)**	**Parental education effect on mediator (path a, X M)**			**Mediator effect on BMI** **(path b, M Y)**			**Direct effect** **(path c', XY**_**adjM**_**)**			**Indirect effect** (a*b, XMY)	
**Total effect (path c) (95% CI):** **−0.25 (−0.40;−0.09)**										
**Mediators**	**ß**	**95% CI**	***p-*** **value**	**ß**	**95% CI**	***p-*** **value**	**ß**	**95% CI**	***p-*** **value**	**ß**	**95% CI**
Breakfast consumption (days/week)	**0.08**	0.01;0.15	0.018	**−0.36**	−0.56,−0.15	<0.001	**−0.22**	−0.37;−0.07	0.005	**−0.03**	−0.07;−0.01
Sugar sweetened beverages (glasses/week)	**−1.38**	−1.94;−0.82	<0.001	0.01	−0.1;0.04	0.259	**−0.22**	−0.39;−0.07	0.005	−0.02	−0.05;0.01
Total screen time (hours/week)	**−2.17**	−2.96;−1.38	<0.001	**0.03**	0.01;0.04	0.004	**−0.19**	−0.35;−0.03	0.018	**−0.06**	−0.10;−0.02
Physical activity (days/week)	0.05	−0.02;0.12	0.179	−0.12	−0.33;0.09	0.271	**−0.24**	−0.39;−0.09	<0.001	−0.01	−0.02;0.01
Mental health problems (SDQ, total score)	**−0.23**	−0.39;−0.07	0.005	0.03	−0.06;0.12	0.513	**−0.24**	−0.40;−0.09	0.003	−0.01	−0.04;0.02
Health-related quality of life (KIDSCREEN-10)	0.15	−0.18;0.48	0.382	−0.02	−0.07;0.02	0.275	**-0.25**	−0.40;-0.09	0.002	−0.01	−0.03;0.01

*Path a, association between parental education and the mediator; path b, association between mediator and BMI adjusted for parental education; total effect, unadjusted association between parental education and BMI; direct effect, association between parental education and BMI adjusted for the mediator; indirect effect, product of path a and path b (bootstrapping, 5,000 samples). X, Predictor (Parental education); Y, Outcome (BMI); M, Potential mediator; , unstandardized regression coefficient; significant effects in bold*.

There was a significant effect of parental education on several potential mediators (path a). Parental education was positively associated with breakfast consumption (ß: 0.08; CI: 0.01; 0.15) (i.e., lower parental education, less regular breakfast consumption) and negatively associated with consumption of sugar-sweetened beverages (ß:−1.38; CI:−1.94;−0.82), total screen time (ß:−2.17; CI:−2.96;−1.38) and mental health problems (ß:−0.23; CI:−0.39;−0.07) (i.e., lower parental education, higher consumption of sugar-sweetened beverages, longer total screen time and more mental health problems). There was neither a significant association between parental education and physical activity nor parental education and HRQoL.

Path b, the association between a potential mediator and BMI adjusted for parental education, showed two significant effects. Breakfast consumption had a negative (ß:−0.36; CI:−0.56;−0.15) and total screen time a positive effect (ß: 0.03; CI: 0.01; 0.04) on the BMI.

Both potential mediators also showed an indirect effect (breakfast consumption: ß:−0.03; CI:−0.07;−0.01; total screen time: ß:−0.06; CI:−0.10;−0.02). As the direct effect was also significant (breakfast consumption: ß:−0.22; CI:−0.37;−0.07; total screen time: ß:−0.19; CI:−0.35;−0.03), breakfast consumption and total screen time can separately be identified as partial mediators.

#### Multiple Mediator Models

**Figure 3:**
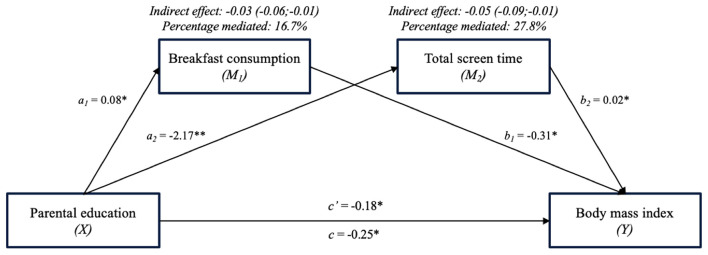
The mediating effect of breakfast consumption and total screen time on the association between parental education and BMI (Multiple mediator model 1). *p < 0.05.

**Figure 4:**
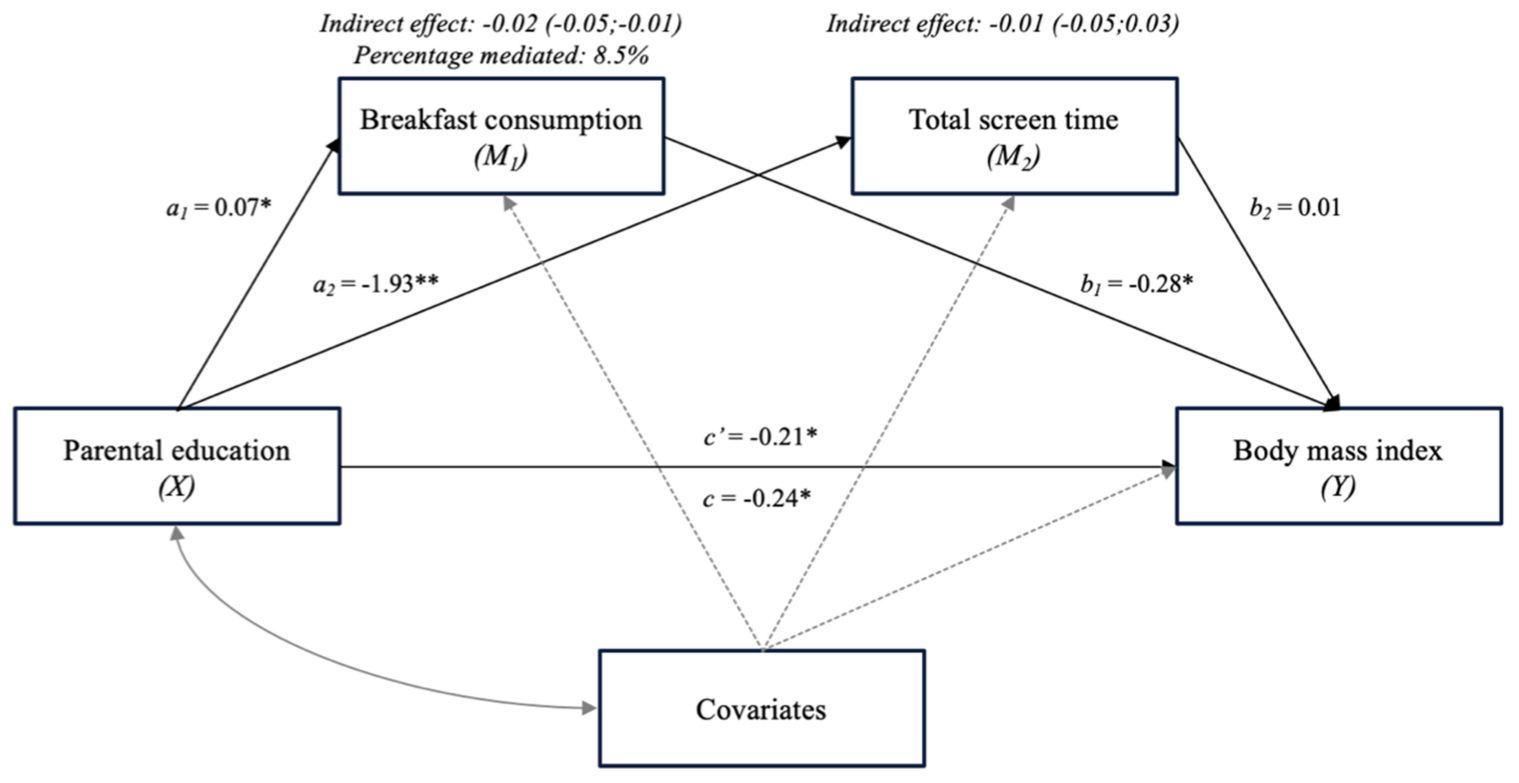
The mediating effect of breakfast consumption and total screen time on the association between parental education and BMI, adjusted for covariates (age, gender, interaction of age × gender, migration status) (Multiple mediator model 2). *p < 0.05.

The results concerning the multiple mediation models are presented in Figures 3, 4. The two identified mediators with significant indirect effects in the single mediation model were included in the multiple mediation models.

Results from multiple mediator model 1 (see Figure 3) are comparable to the results of the single mediation models, as both breakfast consumption and total screen time were identified as partial mediators again. Breakfast consumption mediated 16.7% and total screen time 27.8% of the association between parental education and BMI.

In multiple mediator model 2 (see Figure 4), the analysis was adjusted for the covariates age, gender, interaction of age × gender, and migration status. The effect sizes of the previously identified mediators decreased and total screen time showed no significant indirect effect on the association of parental education and BMI anymore. Breakfast consumption mediated 8.5% after adjusting for the covariates. In Table 3 the effects of the covariates on the multi mediation model are presented. Gender had a significant effect on breakfast consumption (ß:−0.50; CI:−0.80,−0.18), age had a significant effect on total screen time (ß: 4.81; CI: 1.88, 7.75) and on BMI (ß: 1.05; CI: 0.45, 1.66). Furthermore, the interaction of age and gender (ß:−0.41; CI:−0.79,−0.04) and the migration status (ß:−1.55; CI:−2.97,−0.13) had significant effects on BMI. In the total effect model age (ß: 1.12; CI: 0.52; 1.72), the interaction of age and gender (ß:−0.42; CI:−0.80,−0.04) and migration status (ß:−3.16; CI:−3.16,−0.33) had significant effects on BMI.

**Table 3:** Effects of the covariates in the multiple mediator model (Multiple mediator model 2).

**(*n* = 460)**	**Breakfast consumption (M)**			**Total screen time (M)**			**BMI (Y)**			**Total effect**		
**Covariates**	**ß**	**95% CI**	***p-*value**	**ß**	**95% CI**	***p-*value**	**ß**	**95% CI**	***p-*value**	**ß**	**95% CI**	***p-*value**
Age (in years, T0)	−0.15	−0.42;0.12	0.276	**4.81**	1.88;7.75	0.001	**1.05**	0.45;1.66	0.001	**1.12**	0.52;1.72	<0.001
Gender	**−0.50**	−0.80;−0.18	0.002	−0.09	−3.39;3.60	0.960	−0.50	−1.05;0.40	0.166	−0.36	−1.06:0.34	0.316
Interaction age × gender	0.02	−0.15;0.19	0.827	−0.46	−2.32;1.39	0.622	**−0.41**	−0.79;−0.04	0.032	**−0.42**	−0.80;−0.04	0.030
Migration status (T0)	0.57	−0.06;1.20	0.077	−6.09	−13.0;0.81	0.083	**−1.55**	−2.97;−0.13	0.032	**−1.75**	−3.16;−0.33	0.016

*T0, Baseline assessment; X, Predictor (Parental education); Y, Outcome (BMI); M, Potential mediator; C, covariate; ß, unstandardized regression coefficient; significant effects in bold*.

### Sensitivity Analyses

Sensitivity analyses were performed to compare the results (i) with and without missing data imputation and (ii) using raw BMI scores and BMI percentiles as the outcome. Firstly, no substantive differences between imputed and original data were found (results not presented in detail). Secondly, using BMI percentiles, results of the single mediator models were mostly robust. The total effect from parental education to BMI percentiles (ß:−1.99; CI:−3.15,−0.84), the indirect effect via breakfast consumption (ß:−0.15; CI:−0.38,−0.003) and the non-significant associations of the other potential mediators (i.e., consumption of sugar-sweetened beverages, physical activity, mental health problems, HRQoL) and BMI were confirmed. In contrast, the indirect effect via total screen time (ß:−0.19; CI:−0.50, 0.11) was not significant now (results not presented in detail).

## Discussion

In this longitudinal study, the direct and indirect paths of behavioral and psychological factors on the association between parental education and the BMI of children and adolescents were examined using data from a large population-based sample from Germany. The results show that the parental education level is inversely associated with the BMI of children and adolescents 5 years later. A lower level of parental education is linked to skipping breakfast, higher consumption of sugar-sweetened beverages, longer total screen time, and a higher amount of mental health problems. Children and adolescents, who skipped breakfast and had a longer total screen time, had a higher BMI 5 years later. Partial mediators of the association between parental education and the BMI were breakfast consumption and total screen time. After adjusting control variables, only breakfast consumption remained significant. The consumption of sugar-sweetened beverages, the duration of physical activity, the amount of mental health problems, and the level of HRQoL had no mediating effect on the association between parental education and BMI.

The first *hypothesis*, i.e., a lower level of parental education is associated with a higher BMI in children and adolescents 5 years later, was confirmed by this study. Other longitudinal and cross-sectional studies reported similar findings. The systematic review by Shrewsbury and Wardle (28), which compared studies examining socioeconomic status and adiposity in childhood, showed that 75% of the analyzed studies using parental education as an indicator for socioeconomic status found an inverse relationship between parental education and obesity. Recently, a systematic review of longitudinal studies was published, also confirming the relationship between a low parental educational level and an increased BMI (63). For future research it seems promising to disentangle the separate and interactive effects of maternal and paternal educational level on their children's BMI.

The second *hypothesis*, i.e., a lower parental educational level is associated with behavioral and psychological risk factors, was only partially substantiated in this study. A lower parental education level predicted skipping breakfast, higher consumption of sugar-sweetened beverages, longer total screen time, and a high amount of mental health problems. The results are in line with previous studies. In several European countries, children of lower educated parents were more likely to skip family breakfast than children of more educated parents (64). Furthermore, evidence that children of mothers with a low level of education consume more sugar-sweetened beverages than children of mothers with a high level of education was found by a study among 11-year-old children in Norway (65). An inverse relationship between low parental education and longer screen time was found in a study carried out in Norway (66). A systematic review of socioeconomic inequalities and mental health problems confirmed the association between lower parental education and more mental health problems (67).

However, in our study, parental education did not predict the duration of physical activity or the level of quality of life. Gustafson and Rhodes found indefinite results in their review of the relationship between parental education and physical activity (68). The authors explain the findings by a large number of confounders that can occur when measuring physical activity. This could also explain why parental education did not predict physical activity in the presented study. The association between parental education and HRQoL was also investigated by Rueden et al. (69). However, they used the KIDSCREEN-52 questionnaire, which measures nine different dimensions of HRQoL, instead of the KIDSCREEN-10, which measures a total HRQoL index. The results of Rueden's et al. study showed that only one of the nine dimensions, i.e., physical wellbeing, was related to parental education.

The *third hypothesis*, i.e., more pronounced risk factors cohere with a higher BMI 5 years later, could only be confirmed for breakfast consumption and total screen time. There was no significant longitudinal effect of consumption of sugar-sweetened beverages, physical activity, mental health problems, or HRQoL on BMI. The longitudinal association between breakfast consumption and BMI was also found by a review in which 80% of the longitudinal studies examined showed that regular breakfast consumption was associated with a reduced risk of overweight and obesity in children (70). The positive relationship between total screen time and BMI could also be confirmed by a longitudinal study, conducted in the USA, which found an association between longer screen time and higher BMI (69). Another study states that reducing screen time in adolescence and into adulthood can be a promising strategy for reducing the prevalence of obesity (71).

Existing literature reports mixed results concerning the longitudinal effect of the consumption of sugar-sweetened beverages and overweight or obesity. A meta-analysis investigating the effect of the consumption of sugar-sweetened beverages on weight gain found the association to be close to zero (72). However, other studies found a significant effect on the consumption of sugar-sweetened beverages and increase of BMI (73, 74). An explanation for these heterogeneous results could be different types of measurements that were used to determine the number of sugar-sweetened beverages consumed. Furthermore, the use of self-reported data could lead to bias as it can be affected by social desirability, especially for those who are already overweight, or the growing awareness of the harmful effects of sugar-sweetened beverage's consumption. Studies that investigated the longitudinal effect of physical activity and BMI showed inconsistent results. In a longitudinal intervention study in the Czech Republic, physical activity had a vital role in reducing overweight and obesity among children (75). Similar results were found by a systematic review investigating the associations between objectively physical activity and adiposity in children and adolescents (76). However, two other studies showed no significant association between physical activity and BMI (71, 77). These mixed results could be due to different ways of measuring physical activity. An epidemiological study by Hestetun, Svendsen, and Oellingrath found mixed evidence on the association between overweight and mental health problems in 12- to 13-year-old Norwegian children (78). Reasons for the indefinite association could be the low number of children with a high amount of mental health problems in this study as well as in our study. Further studies should investigate the association in a sample of children with more pronounced mental health problems. As far as we know, HRQoL has only been investigated as a result of BMI (79–81) and not as a predictor for BMI. Therefore, the results of our study cannot be compared to previous literature. More research is needed for investigating HRQoL as a predictor of overweight and obesity.

The *fourth hypothesis*, parental education has an indirect effect via breakfast consumption, consumption of sugar-sweetened beverages, total screen time, physical activity, mental health problems and HRQoL on the child and adolescent's BMI, was only validated for two of the risk factors. In line with previous studies, the presented study showed significant indirect effects of breakfast consumption and total screen time on the association of parental education and BMI of children and adolescents. Gebremariam et al. (44) review of mediation effects on the link between socioeconomic status (parental education) and obesity, also showed indirect effects of energy balance-related behaviors such as breakfast consumption, television and computer use. In addition, this review confirms the indeterminate role of physical activity, which could be due to the self-reported measurement of physical activity. As this is the first study on the mediating effects of mental health problems and HRQoL on the association of parental education and BMI, further studies are needed to compare results.

The proportion explained by the mediators in the unadjusted model was moderate, with the direct effect remaining significant after adjustment for the mediators. These results imply that other factors not included in the analyses may play a mediative role in the relationship between parental education and child body composition. Future analyses should compare further mediators, as this may increase knowledge of the complex interplay between parental education and child body composition. In addition to the investigated physical and psychological health behaviors of adolescents, studies have shown parental factors as a pathway between parental education and BMI, such as the parent's BMI, parental psychopathology, their working hours, income along with their own health behaviors (82), and their awareness, resources, and abilities to care for their child's health (83). Further promising pathways seem to be parent-child interaction [i.e., attachment, parenting style, neglect (82, 84)], early childhood factors [i.e., breastfeeding, birth weight (85)] and other determinants, such as child-care attendance (82), and critical life events (86). Genetic predispositions to increased BMI can also act as a pathway, since those with higher educational attainment may have a lower genetic risk score for an increased BMI (87).

After adjusting for important control variables as age, gender, and migration status or using age- and gender-specific BMI percentiles in the sensitivity analysis, it is shown that the previously significant mediator, total screen time, no longer shows a significant indirect effect and the effect of breakfast consumption decreases. The different effects of age, gender, and migration status indicate there are different pathways of how these risk factors mediate the association between parental education and BMI. Similar effects caused by gender, age, and migration have already been found in previous studies (28, 88–90). These effects and their specific pathways should be further investigated to be able to develop tailored intervention and prevention strategies.

### Strengths and Limitations

When interpreting the results of this study, the following strengths and limitations must be taken into account. The strengths of the studies include the data of the study, which is a partial sample of the BELLA study. The BELLA study is one of the most important longitudinal studies for assessing mental health problems in a population-based sample of children, adolescents, and young adults in Germany. The strengths of the BELLA study lie in the large sample size and the longitudinal timeline, which makes it possible to examine the participants over time. By measuring weight and height during follow-up physical examination, the BMI was calculated based on measured data. This decreases biases that can be caused by a self-reporting weight and height (91). A further advantage of this study is the multiple included behavioral factors as well as psychological factors, which were measured using internationally comparable methods. To the best of our knowledge, this study is the first to examine both behavioral factors and psychological factors on a longitudinal level.

The present study has the following limitations. First, breakfast consumption, consumption of sugar-sweetened beverages, physical activity, and total screen time were assessed in a retrospective self-report. These data are responsible for memory and may suffer from social desirability, which can lead to a bias in the data. Future studies should measure these variables in more objective ways, such as in a clinical setting, documentation in a diary or on digital devices as well as on a longitudinal level. Furthermore, information on the nutritional content of the food consumption variables could be recorded. Although the BMI is a globally established measurement for determining the body composition and is therefore internationally comparable; nevertheless, the BMI has some disadvantages: (i) The proportions of fat and muscle mass are not taken into account and thus, for example, athletes with low fat and high muscle mass, can have a higher than normal BMI and erroneously being categorized as overweight (92); (ii) Yet, BMI percentiles for adolescents and young adults cannot be compared without caution; (iii) To conclude whether someone is (un)healthy or healthier than another more parameters than height and weight would be necessary, e.g. blood parameters (glucose, lipids) or muscle percentage. Finally, there might be unobserved heterogeneity which drive both children's BMI and parental education for what reason causality of effects cannot be claimed. Future studies should measure children's BMI at least twice.

### Implications

Based on previous research, the prevention of overweight and obesity among children and adolescents from families with a higher educational level is more likely to succeed (93–95). However, the results of this study show that children from parents with lower education are more affected by a higher BMI, which can lead to overweight and obesity. Therefore, when developing strategies to reduce the increase of the BMI in children and adolescents, particular attention should be paid to target groups with lower parental educational levels in order to improve effectiveness. Prevention should include measures that reach and are accepted by these groups in particular in addition to scientifically proven ways for prevention of overweight and obesity in general, independently of parental education level. The aim is to support parents and children in changing their breakfast habits toward a regular breakfast every school day and to promote reduced total screen time through improved awareness and information as the results of this study show these factors to be mediating the association between parental education and BMI. To achieve this, measures must be taken at different levels; at the individual level, involving children and their parents, at the school level and with the involvement of politics and society.

## Conclusion

This study shows that prevention measures should be particularly targeted at children and adolescents of parents with low levels of education to address the significant public health challenge of increased BMI. Tailored strategies to prevent the development of overweight and obesity in the lower educated population among children and adolescents should develop specific messages focusing on the importance of promoting daily breakfast consumption at home and reducing screen time. Further studies are needed to investigate tailored development, implementation, and evaluation of prevention measures.

## Data Availability Statement

The original contributions presented in the study are included in the article/Supplementary Materials, further inquiries can be directed to the corresponding author/s.

## Ethics Statement

The studies involving human participants were reviewed and approved by the Ethics Committee of the University Hospital Charitè in Berlin, Germany, and the Federal Commissioner for Data Protection in Germany. Written informed consent to participate in this study was provided by the participants' legal guardian/next of kin. Written informed consent was given by the participants' parents (if aged 7-17) and additionally by the participants themselves (aged 14 or older).

## Author Contributions

TS, A-KM, MR, AS, and UR-S contributed to the conception and design of the study. TS and A-KM organized the database. TS performed the statistical analysis in close consultation with A-KM and MR. AS provided special expertise concerning BMI and overweight. TS wrote the first draft of the manuscript. A-KM wrote sections of the manuscript. UR-S provided resources and conducted project administration and supervision. All authors contributed to manuscript revision, read, and approved the submitted version.

## Funding

The German Federal Ministry of Health (BMG) funded the BELLA assessment from 2009 to 2012.

## Conflict of Interest

The authors declare that the research was conducted in the absence of any commercial or financial relationships that could be construed as a potential conflict of interest.

## Publisher's Note

All claims expressed in this article are solely those of the authors and do not necessarily represent those of their affiliated organizations, or those of the publisher, the editors and the reviewers. Any product that may be evaluated in this article, or claim that may be made by its manufacturer, is not guaranteed or endorsed by the publisher.
