# Supplementary material for: Pathways of Parental Education on Children's and Adolescent's Body Mass Index: The Mediating Roles of Behavioral and Psychological Factors
Source: Front Public Health. 2022 Mar 7;10:763789. doi: 10.3389/fpubh.2022.763789 (PMC8936576; doi:10.3389/fpubh.2022.763789)
Supplement: Supplementary file 1 [file Table_1.DOCX]

**Supplementary Table 1.** Comparison of the analyzed study sample and children and adolescents only participated at baseline.

|  | | | **Study sample**  (*n* = 460) | | | **Children and adolescents only participated at T0**  (*n* = 822) | | | **Significance test and effect sizes of differences**  paired samples t-test/χ²-test | |  |  |
| --- | --- | --- | --- | --- | --- | --- | --- | --- | --- | --- | --- | --- |
|  | | | ***n*** | **Valid %** | ***M* (*SD*)** | ***n*** | **Valid %** | ***M* (*SD*)** | **Cohen’s d/Cramer’s V** | | |  |
| Gender | | |  |  |  |  |  |  |  | .030 ^n.s..^ | |  |
| *Male* | | | 218 | 47.4 |  | 415 | 50.5 |  |  |  | |  |
| *Female* | | | 242 | 52.6 |  | 407 | 49.5 |  |  |  | |  |
| Age (in years) | | |  |  |  |  |  |  |  |  | |  |
| *T0 (11-17 years)* | | | 460 |  | 14.00 (1.84) | 822 |  | 14.76 (1.72) |  | -.432^*^ | |  |
| *T1 (15-23 years)* | | | 460 |  | 18.66 (1.92) | - |  | - |  | - | |  |
| Migration status (T0) | | |  |  |  |  |  |  |  | .009 ^n.s.^ | |  |
| *Yes* | | | 30 | 6.5 |  | 50 | 6.1 |  |  |  | |  |
| *No* | | | 430 | 93.5 |  | 772 | 93.9 |  |  |  | |  |
| Parental education (in years, T0) | | | 459 |  | 13.77 (2.34) | 818 |  | 13.48 (2.42) |  | .115 ^n.s.^ | |  |
| BMI (kg/m2) (T1) | | | 460 |  | 22.61 (3.98) | - |  | - |  | - | |  |
| Breakfast consumption (in days per weekdays, T0) | | | 451 |  | 3.94 (1.76) | 787 |  | 3.61 (1.97) |  | .176^*^ | |  |
| Sugar-sweetened beverages (in glasses per week, T0) | | | 449 |  | 7.24 (14.74) | 783 |  | 7.77 (13.65) |  | -.044 ^n.s.^ | |  |
| Total screen time (in hours per week, T0) | | | 451 |  | 38.71 (20.81) | 787 |  | 42.62 (21.99) |  | -.181^*^ | |  |
| Physical activity (in days per week, T0) | | | 449 |  | 3.93 (1.78) | 784 |  | 3.75 (1.83) |  | .100 ^n.s.^ | |  |
| Mental health problems (SDQ total score, T0) | | | 405 |  | 9.47 (4.39) | 747 |  | 9.29 (4.39) |  | .039 ^n.s.^ | |  |
| HRQoL (KIDSCREEN-10, T0) | | | 423 |  | 52.03 (8.72) | 715 |  | 51.42 (8.84) |  | .070 ^n.s.^ | |  |
| *Note*. T0 = Baseline assessment; T1 = Follow-up; SDQ = Strengths and Difficulties Questionnaire (51); KIDSCREEN-10: HRQOL Index (53). *M* = mean; *SD* = standard deviation; - = not assessed/applicable; ^n.s.^ = non-significant difference, * p < .05 (*p* was Bonferroni-Holm corrected). | | | | | | | | | | | | |
|  |  |  |  |  |  |  |  |  |  |  |  |  |
